# Governing Evidence in Contentious Times: A GUARD Framework for Public Health Leadership

**DOI:** 10.3389/phrs.2026.1609645

**Published:** 2026-04-17

**Authors:** Geneviève Chene, Marie Preau, Paulette Lenert, Yves Martin-Prevel, Roch Giorgi

**Affiliations:** 1 Université de Bordeaux, Bordeaux, France; 2 Centre Hospitalier Universitaire de Bordeaux, Talence, France; 3 Inserm-U1219, Bordeaux, France; 4 Universite Lumiere Lyon 2 Institut de Psychologie, Bron, France; 5 Inserm U1290, Lyon, France; 6 Luxembourg Chamber of Deputies, Luxembourg, Luxembourg; 7 IRD, Institut de recherche pour le developpement, Marseille, France; 8 Universite de Montpellier, UMR MOISA, Montpellier, France; 9 Aix-Marseille Univ, SESSTIM, ISSPAM, Marseille, France; 10 Assistance Publique—Hopitaux de Marseille, Marseille, France; 11 INSERM U-1252, Marseille, France

**Keywords:** accountability, decision making, evidence governance, health equity, leadership, credibility, infodemic management, legitimacy

## Abstract

Public health leadership faces widening inequities, uneven life expectancy trends, and growing information disorder. Experience from COVID-19 showed that shortcomings often stem less from lack of evidence than from weaknesses in how evidence inform decisions. This commentary reframes the challenge as one of evidence governance: ensuring that knowledge is interpreted, debated, and translated into fair, accountable action. We introduce GUARD, a practice-oriented framework for governing evidence: Govern in public, User power-sharing, Architect and audit integrity, Resist manipulation of meaning, and Demonstrate legitimacy. Rather than proposing new principles, GUARD operationalizes existing ones into implementable governance routines, offering public health leaders a practical pathway to strengthen legitimacy, reduce inequities, and sustain trustworthiness under uncertainty.

## Introduction

Across many settings, life expectancy is evolving unevenly, stalling or declining in some populations while improving in others, at a time when inequities are widening and societies face overlapping crises, from pandemics to climate disruption to polarization and disinformation. COVID-19 showed that even sophisticated surveillance and modeling do not guarantee equitable or effective action. What often fails is not evidence itself, but how evidence is governed: how information is made visible, interpreted, communicated, and translated into decisions that allocate benefits, burdens, and protections.

The central challenge for public health leadership today is no longer generating evidence, but governing how evidence is interpreted, debated, and translated into fair and accountable actions. Trust, a social relationship, cannot be commanded; trustworthiness, an institutional quality, must be demonstrated. This commentary shifts attention from *evidence generation* to *evidence governance,* what counts as evidence, how uncertainty is handled, whose knowledge is included, and how decisions are justified, corrected, and repaired, so institutions can earn public confidence and, over time, public trust.

We introduce GUARD, a practice-oriented approach for governing evidence, particularly in contentious times. GUARD is a minimum-viable governance operating framework that turns principles of trust, equity, and evidence use into routines leaders can implement, audit, and adapt under pressure, including in low-resourced settings.

## What This Commentary Adds

Recent strands of work provide key foundations for understanding evidence use in public health decision-making. Trust-focused analyses emphasize institutional legitimacy [1], while Parkhurst’s work on the good governance of evidence highlights relevance, rigor, and democratic accountability [2]. More recent integrated models of evidence-informed policymaking emphasize combining multiple forms of evidence with contextual judgement [3]. Governance frameworks such as TAPIC (Transparency, Accountability, Participation, Integrity, policy Capacity) define core principles for decision-making and implementation [4]. Within this evolving field, our previous work extended these discussions by highlighting the importance of equity-oriented perspectives and disaggregated data to make uneven harms visible [5].

Together, these strands clarify why governing evidence matters but less often specify how leaders can operationalize accountability, shared decision rights, and corrective learning within existing mandates. GUARD does not introduce new governance principles: it rather translates established ones into practical, repeatable routines applicable when evidence is contested and harms emerge unevenly.

Within this approach, we distinguish *evidence-informed policy* from *data-driven analytics*. Good policy integrates quantitative, qualitative, and experiential inputs while acknowledging value trade-offs [4]. This commentary is written for senior public health leaders responsible for co-governing data and legitimacy. The following sections operationalize this approach through five interconnected leadership practices that move from public reasoning to legitimacy and repair.

## G-Govern in Public: From Openness to Public Accountability

Transparency begins before publication: why data are collected, whose interests they serve, and how communities can shape their use. It requires a public account of data provenance and limitations [6], and communication of uncertainty and value choices. Yet visibility alone does not secure justice. Disaggregated reporting prevents average effects from masking uneven harms, underscoring that indicators and categories are governance choices, not neutral technical steps, that determine whose lives and risks are prioritized.

Accountability builds on transparency and answerability by requiring scrutiny, correction, and remedy: explaining what was done and why, then being held responsible for outcomes through evaluation and repair. It thrives when institutions invite evaluation and when communities can trigger review and redress.

*Governance lever*: For major decisions, publish a one-page public note stating the evidence used, key assumptions and uncertainties, expected (disaggregated) impacts, and triggers for revision with named responsibility.

## U-User Power-Sharing: Shared Power, Not Consultation

Partnership must mean shared power, not symbolic consultation. Trust grows when affected communities shape priorities, acceptable uses, and oversight [1]. Participatory research offers one pathway [7], but durable co-stewardship requires mechanisms that transfer authority, not just voices. Representation rules, compensation for participation, and conflict of interest safeguards reduce the risks of tokenism and elite capture. Co-produced fieldwork can strengthen participation and relevance, including face-to-face data collection in marginalized groups [8].

Shared power also depends on shared understanding. Data literacy, within institutions and across communities, is integral to meaningful participation; participation, in turn, deepens literacy through practice and feedback. Leaders should democratize skills for interpreting and questioning evidence so new technologies expand inclusion rather than reinforce inequity.

*Governance lever*: Establish standing community evidence councils (or stewardship boards) with written decision rights: approving priority questions and data uses, reviewing subgroup impacts, and triggering review when harms are detected.

## A-Architect and Audit Integrity by Design

Power-sharing endures only when safeguards are enforceable. Integrity therefore begins with protection: privacy-preserving architectures, role-based access, and bias controls must be designed from the outset. De-identification alone is insufficient because, while it removes direct identifiers, it does not correct imbalances in data composition or the social patterns embedded in datasets; machine-learning models trained on de-identified but unrepresentative data can still reproduce existing inequalities and amplify inequities.

Public health leaders should treat AI and digital tools as social as well as technical systems, because their design and deployment influence whose risks are prioritized and how benefits and burdens are distributed; governance must therefore address social consequences and accountability, not only technical performance. Procurement and vendor contracts should require testing model performance across relevant population subgroups (e.g., age, sex, socioeconomic or other contextually relevant categories) where feasible, along with public mitigation plans and explicit rights to audit and withdraw systems that fail to meet standards. Because data sources themselves carry bias, convenience data from platforms, wearables, or social media, often over-representing the most connected rather than the most exposed, should be used only with clearly stated limitations and triangulated against representative samples [9].

Integrity also depends on verification and learning, not assertion. Independent audits, public registries for models and indicators, and enforceable remediation timelines make safeguards actionable. Digital tools and AI should be treated as hypotheses and tested in real-world conditions for effectiveness, equity, acceptability, and environmental footprint. Time-limited pilots and sunset clauses requiring re-evaluation before continued use help prevent premature scaling. Public reporting of null and negative results, protection of evaluators from political or commercial interference, and transparent course correction are equally essential for sustaining scientific rigor and public trust.

*Governance lever*: Make auditability a procurement gate; if a tool cannot be audited, stress-tested for subgroup harms, and withdrawn, it should not be deployed.

## R-Resist Manipulation of Meaning: Credibility and Infodemic Governance

Even ethical systems can fail if meaning is distorted or weaponized. In an age of polarization and industrial-scale misinformation, governance of evidence must therefore include governance of communication and not political messaging, but stewardship of information quality so public can assess claims, uncertainty, and trade-offs.

Evidence does not make choices, people do. Policy is where evidence meets values. Crises require explicit deliberation about dignity, solidarity, and fairness [10]. Leaders can normalize structured public dialogue, such as citizen juries or assemblies, that allow collective examination of evidence, and publish brief records describing how public values and equity considerations were weighed alongside effect sizes, costs, feasibility, and disaggregated impacts.

WHO’s infodemic management guidance highlights practical capacities: social listening, pre-bunking and debunking, rapid rumours review, and co-created messages with trusted community figures [11], paired with plain-language communication that conveys empathy and uncertainty honestly, alongside transparent corrections when needed. Institutions should also protect experts who speak publicly, so scientific voices can contribute without harassment or politicized reprisal. Trust also requires long-horizon investment in media and data literacy, starting in early life. Finland’s curriculum-wide model illustrates literacy as civic infrastructure beyond the health sector.^1^

*Governance lever*: Publish an updated protocol specifying what triggers the update, who approves it, and how corrections are communicated within a defined timeframe, confronting the partisan distortion or selective use of evidence without retreating into expert-only decision-making.

## D-Demonstrate Legitimacy: Close the Loop From Promise to Repair

GUARD is a practice framework, but legitimacy is the proof. Legitimacy is demonstrated when leaders close the accountability loop: making commitments explicit, tracking real-world impacts (including subgroup effects), acting on what they learn, correcting course promptly, and providing remedy when burdens fall unfairly.

This requires making the full accountability loop visible: publishing the interpretive logic behind policy choices, auditing outcomes against stated aims, and learning from failure constructively—without denigration or scapegoating, while offering remedy to those who bear the greatest burdens.

Legitimacy becomes observable through a small set of markers: routine publication of decision notes and audit logs; documented community decision rights and evidence that they were exercised; timely corrections when evidence shifts; and proof that detected harms trigger mitigation and repair. The test is follow-through: when audits reveal gaps or harms, institutions must show what changed, by whom, and by when.

*Governance lever*: Publish a quarterly public accountability scorecard signed by the responsible authority, reporting actions taken *versus* promised, subgroup impacts, audit findings, corrective actions with named owners and deadlines, and remedies delivered, alongside clear escalation triggers when harms persist.

### Conclusion

The future of public health leadership rests less on new technologies than on the just governance of evidence, practiced through public reasoning, enforceable integrity, and community oversight, so decisions are fairer, harms are prevented, and dignity remains non-negotiable. GUARD helps leaders close the gap between what evidence shows and what institutions do, especially for populations most exposed to harm.

## Footnotes

1 Finnish ministry of education and culture. Media l2025). okm.fi/en/media-literacy [last accessed February 2, 2026].
